# Modal coupling and crosstalk due to turbulence and divergence on free space THz links using multiple orbital angular momentum beams

**DOI:** 10.1038/s41598-020-80179-3

**Published:** 2021-01-22

**Authors:** Zhe Zhao, Runzhou Zhang, Hao Song, Kai Pang, Ahmed Almaiman, Huibin Zhou, Haoqian Song, Cong Liu, Nanzhe Hu, Xinzhou Su, Amir Minoofar, Hirofumi Sasaki, Doohwan Lee, Moshe Tur, Andreas F. Molisch, Alan E. Willner

**Affiliations:** 1 Department of Electrical Engineering, University of Southern California, Los Angeles, CA 90089 USA; 2 King Saud University, Riyadh, 11362 Saudi Arabia; 3 NTT Network Innovation Laboratories, NTT Corporation, Yokosuka, 239-0847 Japan; 4 School of Electrical Engineering, Tel Aviv University, 69978 Ramat Aviv, Israel

**Keywords:** Electrical and electronic engineering, Atmospheric optics, Microwave photonics

## Abstract

Orbital-angular-momentum (OAM) multiplexing has been utilized to increase the channel capacity in both millimeter-wave and optical domains. Terahertz (THz) wireless communication is attracting increasing attention due to its broadband spectral resources. Thus, it might be valuable to explore the system performance of THz OAM links to further increase the channel capacity. In this paper, we study through simulations the fundamental system-degrading effects when using multiple OAM beams in THz communications links under atmospheric turbulence. We simulate and analyze the effects of divergence, turbulence, limited-size aperture, and misalignment on the signal power and crosstalk of THz OAM links. We find through simulations that the system-degrading effects are different in two scenarios with atmosphere turbulence: (a) when we consider the same strength of phasefront distortion, faster divergence (i.e., lower frequency; smaller beam waist) leads to higher power leakage from the transmitted mode to neighbouring modes; and (b) however, when we consider the same atmospheric turbulence, the divergence effect tends to affect the power leakage much less, and the power leakage increases as the frequency, beam waist, or OAM order increases. Simulation results show that: (i) the crosstalk to the neighbouring mode remains < − 15 dB for a 1-km link under calm weather, when we transmit OAM + 4 at 0.5 THz with a beam waist of 1 m; (ii) for the 3-OAM-multiplexed THz links, the signal-to-interference ratio (SIR) increases by ~ 5–7 dB if the mode spacing increases by 1, and SIR decreases with the multiplexed mode number; and (iii) limited aperture size and misalignment lead to power leakage to other modes under calm weather, while it tends to be unobtrusive under bad weather.

## Introduction

The technique of multiplexing multiple data-carrying beams is gaining increasing interest in wireless communications. This is mainly due to its potential to increase the total channel capacity and spectral efficiency in wireless communication links^1–7^. One example of the technique is mode-division-multiplexing (MDM), where multiple data-carrying beams are multiplexed at the transmitter aperture, co-axial propagating through free space, and efficiently demultiplexed at the receiver aperture^1^. Since each beam is chosen as one unique mode from an orthogonal modal basis set, these beams can be efficiently multiplexed at the transmitter (Tx), coaxially propagated, and demultiplexed at the receiver (Rx) with little inherent crosstalk (XT)^1,4,8–10^.

One potential orthogonal modal basis set for MDM could be a subset of Laguerre Gaussian beams carrying orbital angular momentum (OAM)^8^, where each beam has a phasefront that “twists” in a helical fashion as it propagates, and a ring-like intensity profile with a null in the center. Each beam carries OAM, where the OAM value *l* is equal to the number of 2π phase changes along the azimuthal direction in one circle^8^.

OAM beams have been utilized for MDM in both millimeter-wave (mm-wave) and optical systems^1–7^. Previous works have shown that there are multiple fundamental issues in wireless OAM links: (a) the divergence of an OAM beam scales approximately with the square root of $\left|l\right|$ and wavelength. It indicates that an OAM beam with a higher order or/and lower frequency has a larger beam size at the receiver, which leads to higher power attenuation^8^; (b) when an OAM beam propagates through atmospheric turbulence, the power of the transmitted OAM mode is leaked to neighbouring modes, which is proportional to the strength of phasefront distortion *D*/*r*_*0*_, where *D* is the beam size and *r*_*0*_ is the Fried parameter. The value of *D*/*r*_*0*_ increases with mode order and frequency, indicating that an OAM beam with a higher order or/and higher frequency experiences a stronger distortion^11–13^.

Recently, terahertz (THz) wireless communications have attracted increasing attention^15–22^. We note that beam divergence and power attenuation during free-space propagation tend to be the key degrading effects for mm-wave OAM links with carrier waves below 100 GHz^23,24^. However, optical OAM links with carrier waves ~ 200 THz tend to be mainly limited due to the atmosphere-turbulence-induced phasefront distortion^11–13^. Since the THz wave wavelength is shorter than that of mm-wave but longer than that of optical wave, divergence and turbulence effects might degrade THz OAM links in their own manners^14^.

Here, we investigate the fundamental system degrading effects of THz OAM-multiplexing wireless communication links with atmospheric turbulence. We simulate and analyze the effects of divergence, turbulence, limited-size aperture, and misalignment on the signal power and crosstalk of THz OAM links. We find through simulations that faster divergence (lower frequency or/and smaller beam waist) leads to higher power leakage from the transmitted mode to neighbouring modes when the same strength of phasefront distortion *D*/*r*_*0*_ is concerned. However, divergence effect tends to affect much less and the power leakage becomes proportional to the value of *D*/*r*_*0*_ (which is also proportional to frequency, beam waist, and OAM order) when the same atmospheric structure constant *C*_*n*_^*2*^ is concerned. Simulation results show that: (i) the power leakage to the neighbouring mode remains < − 15 dB when we transmit OAM + 4 at 0.5 THz with a beam waist of 1 m through a 1-km link under calm weather (*C*_*n*_^2^ < 5 × 10^–13^ m^−2/3^); (ii) for 3-OAM-multiplexed THz links, the signal-to-interference ratio (SIR) increases by ~ 5–7 dB if the mode spacing increase by 1, and SIR decreases with the multiplexed mode number; and (iii) limited aperture size and misalignment lead to power leakage to other modes under calm weather (*C*_*n*_^2^ < 1 × 10^–13^ m ^−2/3^), while it tends to be unobtrusive under bad weather (*C*_*n*_^2^ = 1 × 10^–11^ m^−2/3^).

## Results

### Concept and simulation model

**Figure 1:**
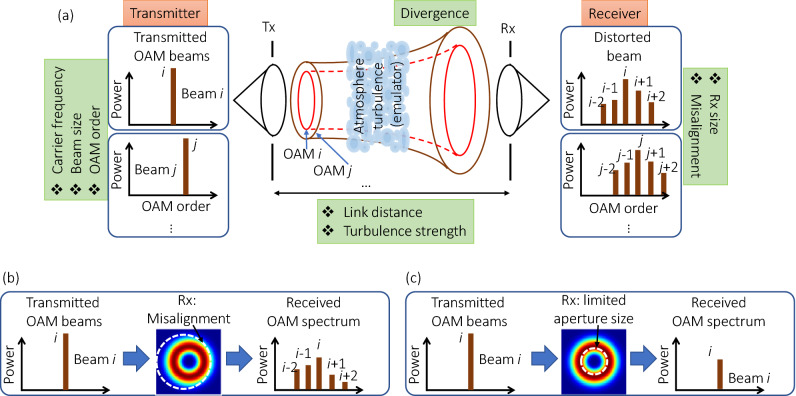
Schematic of a THz communication link using OAM multiplexing. (**a**) Propagation of an OAM beam through turbulence leads to the distortion of its phasefront. At the receiver side, such a beam evolves to a beam with both distorted amplitude and phase profiles. (**b**,**c**) A receiver with limited aperture size and misalignment could further degrade the system performance. *Tx* transmitter, *Rx* receiver.

Figure 1 shows a schematic of a THz communication link using OAM multiplexing. OAM beams diverge and thus lose power when propagating in free space. The strength of divergence depends on many parameters, including frequency, beam size, and OAM mode order^9^. Moreover, the phasefront of a transmitted OAM beam is distorted after propagating through atmospheric turbulence, which results in power leakage to neighbouring modes^12^. The power leakage leads to both power loss and crosstalk among different channels when multiple OAM beams are demultiplexed and detected at the Rx. Besides, a limited receiver aperture size induces additional power loss (Fig. 1b). Finally, misalignment between the transmitter and receiver could further increase power loss and inter-channel crosstalk (Fig. 1c)^10^.

**Figure 2:**
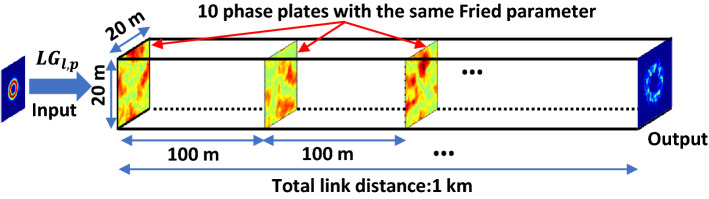
Schematics of the simulation model for studying atmospheric turbulence effects on THz OAM beams. 10 randomly generated phase plates with equal spacing of 100 m are used to emulate the turbulence for a total link distance of 1 km.

We first build a simulation model to emulate atmospheric turbulence; and use it to study the divergence- and turbulence-dependent system-degrading effects on THz OAM links. Figure 2 shows the simulation model for the propagation of THz OAM beams through atmospheric turbulence (see “Methods” for the simulation details). We first emulate atmospheric turbulence by using phase plates, which can be written in *N*-by-*N* matrix forms of random phase distributions according to the Kolmogorov turbulence theory^12,13^. We project each OAM beam onto the 1-st phase plate and subsequentially propagate it in free space, during which it experiences beam divergence. It meets another phase plate, emulating the same turbulence, at a propagation distance of 100 m. We repeat the processes of projection and propagation until a range of 1-km free-space propagation through turbulence is simulated, involving 10 phase plates overall. The key model parameters are listed in Table 1 (see “Methods” for more parameter definitions).

**Table 1:** Parameter definitions.

Parameters	Definitions
*D*	Beam diameter during propagation
*f*	Transmitted beam frequency
*w*_*0*_	Transmitted beam waist
*l*	Transmitted beam OAM order
*C*_*n*_^*2*^	Atmospheric structure constant
*r*_*0*_	Fried parameter

### Influence of OAM beam parameters on XT performance

#### Frequency

**Figure 3:**
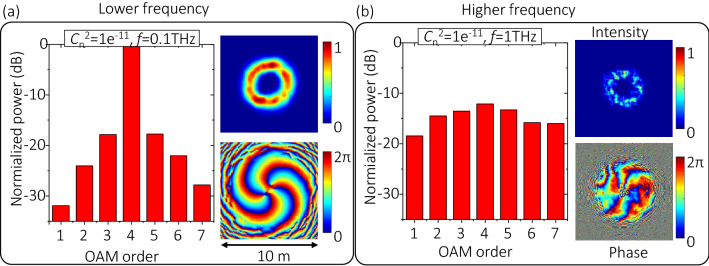
Normalized power distribution on different OAM modes when transmitting OAM + 4 with the same beam waist w0 = 1 m through a 1-km link. Parameters are set as (**a**) ${C}_{n}^{2}$ = 1 × 10^–11^ m^−2/3^, *f* = 0.1 THz, and (**b**) ${C}_{n}^{2}$ = 1 × 10^–11^ m^-2/3^, *f* = 1 THz. A single OAM + 4 beam is transmitted at the transmitter side, and the power distribution on OAM + 2 to + 6 are simulated at the receiver side. The power on OAM + 4 decreases by ~ 12 dB in (**b**), which could be due to the turbulence-induced power leakage from OAM + 4 to other OAM modes.

Wave frequency is one of the key parameters that influence both divergence and turbulence effects. One interesting issue could be exploring how the divergence and turbulence effects degrade the XT performance of THz links with different frequencies. We transmit OAM + 4 with a beam waist of *w*_*0*_ = 1 m through a 1-km link under bad weather (${C}_{n}^{2}$ = 1 × 10^–11^ m^−2/3^). We first investigate through simulations the distortion of the OAM spectrum with two selected frequencies under atmospheric turbulence (Fig. 3). Simulation results show that the 0.1-THz OAM beam is distorted only a little bit (XT to the neighbouring mode is ~ − 15 dB), while the 1-THz OAM beam experience a large distortion effect (XT to the neighbouring mode is ~ − 3 dB). The following might be the reasons why a higher-frequency THz OAM beam gets a higher XT with the same ${C}_{n}^{2}$ (the same atmospheric turbulence): (i) for the cases with the same ${C}_{n}^{2}$, the Fried parameter *r*_*0*_ is proportional to *f* ^6/5^, i.e., ${r}_{0}={(0.423{k}^{2}{C}_{n}^{2}L)}^{-3/5}\propto {k}^{-6/5}\propto {f}^{-6/5}$ (see Eq. (3) in “Methods”). When *f* is varied from 0.1 THz to 1 THz, the value of *r*_*0*_ decreases by a factor of ~ 10 ^6/5^ = 15.8, and the beam diameter decreases by a factor of less than 2 (compare the beam diameter in the intensity profiles in Fig. 3); and (ii) therefore, the strength of phasefront distortion *D*/*r*_*0*_, that an OAM beam experiences at each emulated phase plate during propagation, increases when *f* is varied from 0.1 THz to 1 THz. Thus, a higher frequency leads to higher power leakage to neighbouring modes due to the increase of *D*/*r*_*0*_^12,13^. As shown in Fig. 3b, the atmospheric absorption is not considered. Therefore, the transmitted power is equal to the total received power, i.e., the sum of the received power on all modes (including but not limited to OAM + 1 to + 7). The power on the transmitted OAM + 4 decreases by ~ 12 dB, which could be due to the turbulence-induced power leakage to other modes.

We show the distortion of the OAM spectrum at two selected frequencies as examples. One further step could be to study the dependence of the phasefront distortion on frequency over a broad THz band from ~ 0.1 THz to 10 THz.

**Figure 4:**
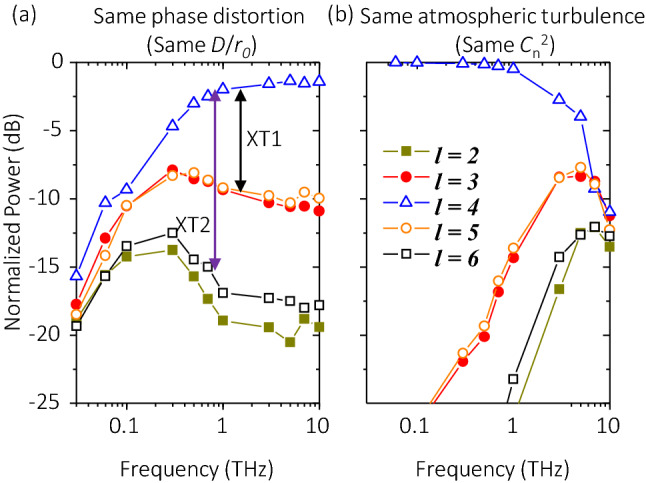
Effects of varying the frequency on normalized power distribution on different OAM modes. (**a**) 200-m link with *D*/*r*_*0*_ = 0.224, *w*_*0*_ = 0.1 m at the transmitter side, (**b**) 200-m link with ${C}_{n}^{2}$ = 1 × 10^–11^ m^-2/3^, *w*_*0*_ = 0.1 m. OAM + 4 is transmitted for all the cases. Legends and scales in *y*-axis in (**a**,**b**) are the same.

We subsequentially investigate the influence of divergence on the XT performance of THz OAM links under turbulence over ~ 0.1–10 THz. In this simulation, we propagate THz OAM beams at different frequencies through emulated phase plates with the same value of the ratio between the transmitted beam size *D* and the Fried parameter *r*_*0*_*.* We refer this condition as the same *D/r*_*0*_***.***Thus, we isolate the influence of *r*_*0*_ , which is frequency-dependent, on the strength of phasefront distortion. We define the power leakage to the right-(higher)-nearest and 2nd-nearest mode as XT1 and XT2, respectively, where the power leakage is normalized by the received power on the transmitted mode. For example, XT1 is the power difference between OAM + 4 (transmitted mode) and OAM + 5 (right-nearest mode); similarly, XT2 is the power difference between OAM + 4 and OAM + 6 (2nd-nearest mode). As shown in Fig. 4a, for a THz link where the input beam has the same value of *D*/*r*_*0*_ at the first emulated phase plate, when frequency goes higher, the signal power on the transmitted OAM mode + 4 increases and the XT to neighouring modes decreases. Specifically, when frequency increases from 0.03 THz to 10 THz for a 200-m link, the signal power increases by ~ 8 dB and XT1 decreases by ~ 5 dB, where the OAM beam has the same value of *D*/*r*_*0*_ = 0.224 at the transmitter side. This might be due to the following: (i) lower-frequency OAM beams diverge faster during free-space propagation, and thus they have larger beam sizes at the same propagation distance; and (ii) the phasefront gets more distorted when the beam size of the transmitted OAM beam increases, which is due to the increase of *D*/*r*_*0*_ during beam propagation^12,13^. These simulation results indicate that when propagating thorough the same phase distortion, THz OAM beams with a higher frequency could experience less modal power leakage and crosstalk.

We further study the dependence of the XT performance on frequency over ~ 0.1–10 THz, regarding both the frequency-dependent divergence and turbulence effects. In this simulation, we propagate THz OAM beams at different frequencies through emulated phase plates with the same atmospheric structure constant *C*_*n*_^2^. We refer this condition as the same *C*_*n*_^2^. Thus, both the beam size and the Fried parameter changes with frequency. In contrast to the case with the same value of *D*/*r*_*0*_ as shown in Fig. 4a, simulation results show that for a THz link where the atmosphere has the same value of ${C}_{n}^{2}$, the higher the frequency, the larger the distortion effect. As shown in Fig. 4b, the signal power decreases by ~ 11 dB and XT1 increases by ~ 38 dB, where the OAM beam propagates through turbulence with the same value of *C*_*n*_^2^ = 1 × 10^–11^ m^−2/3^. This could be due to the same reason explained for Fig. 3, namely, although higher frequency leads to smaller divergence, the value of *D*/*r*_*0*_ still increases with frequency, resulting in larger phasefront distortion. Therefore, in the THz OAM link with the same turbulent atmosphere, there might be a trade-off between the carrier wave frequency and the crosstalk performance.

#### Beam waist

The beam waist is another parameter that influences both divergence and turbulence effects. In general, the beam divergence effect becomes weaker when the beam waist increases. Moreover, increasing the beam waist leads to a larger value of *D*/*r*_*0*_ and thus the phasefront distortion gets stronger under turbulence^12,13^. Therefore, we further study through simulations the dependence of the XT performance on beam waist, when considering the influence of both divergence and turbulence effects. OAM + 4 at *f* = 0.5 THz is transmitted and the link distance is 500 m.

**Figure 5:**
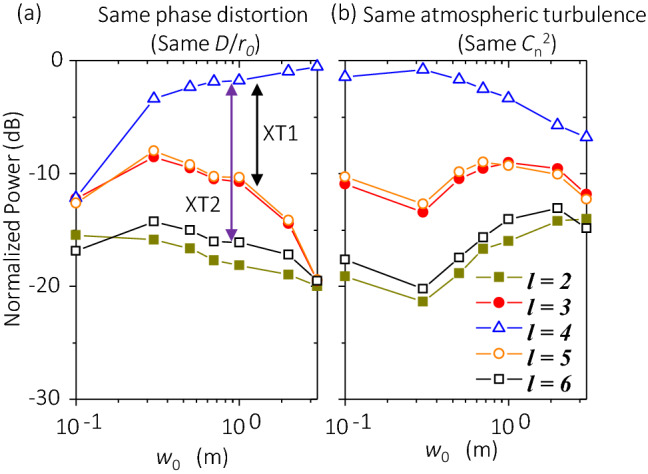
Effects of varying the transmitted beam waist on normalized power distribution on different OAM modes. (**a**) a 500-m link with *D*/*r*_*0*_ = 0.224 at the transmitter side, and (**b**) a 500-m link with ${C}_{n}^{2}$ = 1 × 10^–11^ m^-2/3^. OAM + 4 at *f* = 0.5 THz is transmitted. Legends and scales in *y*-axis in (**a**,**b**) are the same.

Two phenomena can be discerned from Fig. 5. First, for the case with the same *D*/*r*_*0*_ (the same phase distortion), where only the dependence of divergence on the beam waist is considered, a larger beam waist leads to a weaker phasefront distortion. Simulation results show that when the beam waist increases from 0.1 m to 3 m, the signal power increases by ~ 12 dB and XT1 decreases by ~ 20 dB. This might be due to the following: (a) an OAM beam with a larger beam waist diverges slower during propagation; and thus it has a smaller beam size after propagation; and (b) *D*/*r*_*0*_ increases slower during propagation, leading lower power leakage to neighouring modes. Second, in the case with the same ${C}_{n}^{2}$ (the same atmospheric turbulence), we consider the dependence of both divergence and turbulence effects on the beam waist. When we increase the beam waist *w*_*0*_ from 0.1 m to 3 m, the phasefront distortion becomes weaker at first and then becomes larger for *w*_0_ of > 0.3 m. This might be explained by the comprehensive effects of two factors: the beam-waist-dependent divergence effect, and the value of *D*/*r*_*0*_ linearly increases with the beam waist *w*_*0*_. When *w*_*0*_ is < 0.3 m, increasing the beam waist quickly slows down beam divergence. Even though *D*/*r*_*0*_ linearly increases with *w*_*0*_ at the transmitter side, the comprehensive effects still result in a smaller *D*/*r*_*0*_ as the OAM beam propagates. When *w*_*0*_ is > 0.3 m, the phasefront distortion is mainly dependent on the linear relation between *D*/*r*_*0*_ and *w*_*0*_, such that a larger *w*_*0*_ leads to a stronger distortion. In a single-beam THz link, a larger beam waist could lead to some advantages, such as a lower power loss due to smaller divergence effects. However, these results show that there might be an additional concern for THz OAM links with the same turbulent atmosphere, due to that the larger beam waist could lead to higher modal crosstalk.

#### OAM mode order

**Figure 6:**
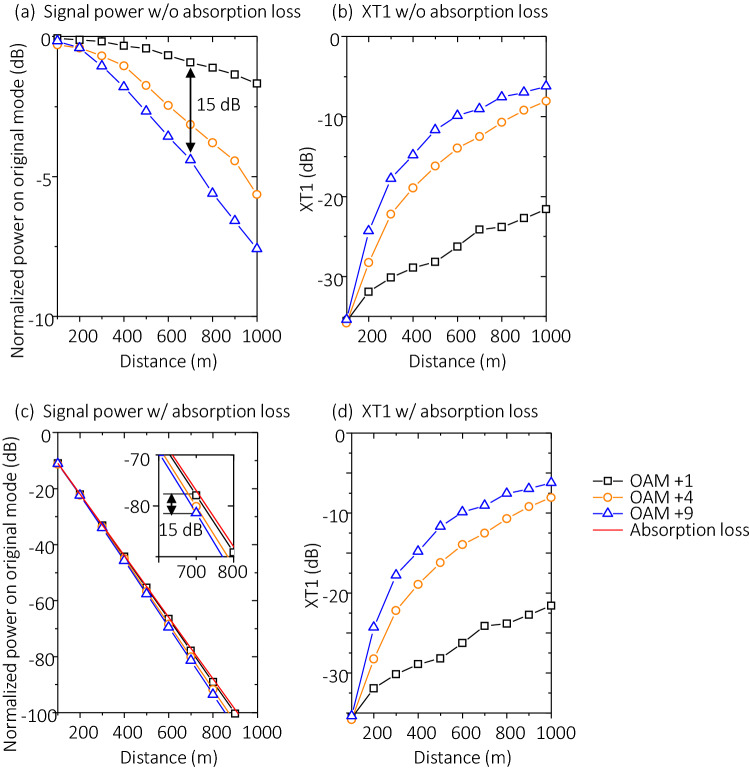
Effects of varying the OAM order on normalized power distribution on different OAM modes. (**a**) The received signal power on the transmitted mode order, and (**b**) XT1 for 1-km link with ${C}_{n}^{2}$ = 1 × 10^–11^ m^-2/3^, *f* = 0.5 THz, and *w*_*0*_ = 0.1 m at the transmitter side without considering the atmospheric absorption. (**c**,**d**) are the corresponding signal power and XT1 of the same link with the atmospheric absorption. The inset in (**c**) shows the signal power at a distance from 600 to 800 m. Different curves are close to each other and thus it might be hard to distinguish the power distribution on different OAM orders. Moreover, comparing the curves in (**b**,**d**) shows that the crosstalk performance seems to be almost the same even when the atmospheric absorption is not considered. The legends in (**a**–**d**) are the same. Scales in y-axis in (**a**–**d**) are different.

One more key parameter that influences the beam divergence effect is the OAM order. In addition, increasing the OAM order also leads to a large value of *D*/*r*_*0*_; Thus, the phasefront distortion becomes stronger. To further explore the OAM-order-dependent system-degrading effects, we investigate through simulations the XT performance when varying the OAM order. Figure 6a,b show the cases when we transmit OAM + 1, + 4, and + 9 with a beam waist *w*_*0*_ = 0.1 m and *f* = 0.5 THz through a 1-km link with ${C}_{n}^{2}$ = 1 × 10^–11^ m^−2/3^.

We compare the cases with and without considering atmospheric absorption loss when varying the OAM order. Instead of building our own modal to consider the absorption, we directly refer to the value of the absorption loss from Ref.^21^. Namely, we firstly use our multiple phase plate model to simulate the power distribution on each OAM mode. We then subtract the power on each OAM mode by the distance-dependent molecule absorption loss. In Ref.^21^, based on the radiative transfer theory together with the information from the HITRA (HIgh resolution TRANsmission molecular absorption) database, the absorption loss is calculated by adding all the molecule absorption. The total absorption loss can be expressed as $Loss={\alpha }_{f}L$, where the frequency-related coefficient ${\alpha }_{f}$ is selected as ~ 0.11 dB/m at 0.5 THz^21^, and *L* is the propagation distance.

Simulation results show that: (i) at the same propagation distance, the higher the OAM order, the lower the signal power and the higher the XT1; and (ii) as the propagation distance increases, the signal power decreases faster while the XT1 increases faster when the OAM order goes higher. Specifically, when the propagation distance increases from 100 m to 1 km without considering the atmospheric absorption, the signal power for OAM + 1, + 4, and + 9 decreases by ~ 1.6 dB, 5.3 dB, and 7.5 dB, respectively, while the corresponding XT1 increases by ~ 14 dB, 27 dB, and 29 dB, respectively. The reason why the higher-order OAM beam becomes more distorted might be explained by the following: (i) for OAM beams with the same beam waist, the beam diameter increases with the OAM order. Therefore, the higher-order OAM beam has a higher value of *D*/*r*_*0*_ at the transmitter side such that it gets a larger distortion; and (ii) the higher-order OAM beam diverges faster so that it has a higher beam size after propagation. Therefore, the value of *D*/*r*_*0*_ increases faster and, thus, the distortion becomes stronger. In addition, Fig. 6c,d show the link performance with atmospheric absorption loss, which is selected as ~ 11 dB per 100-m propagation at 0.5 THz^21^. We note that the signal power decreases by > 100 dB when compared with the 1-km link without considering the atmospheric absorption. However, the differences among the signal power of the different transmitted OAM beams remain almost the same and the XT1 tends to be unchanged. These results indicate that there might be a limitation on the number of OAM modes for THz OAM links with turbulent atmosphere. A THz OAM-multiplexed link could have a higher channel capacity if we multiplex more modes with higher OAM values. However, the channel carrying higher OAM values might experience higher crosstalk and power loss, which could also degrade the system performance.

#### OAM multiplexing

**Figure 7:**
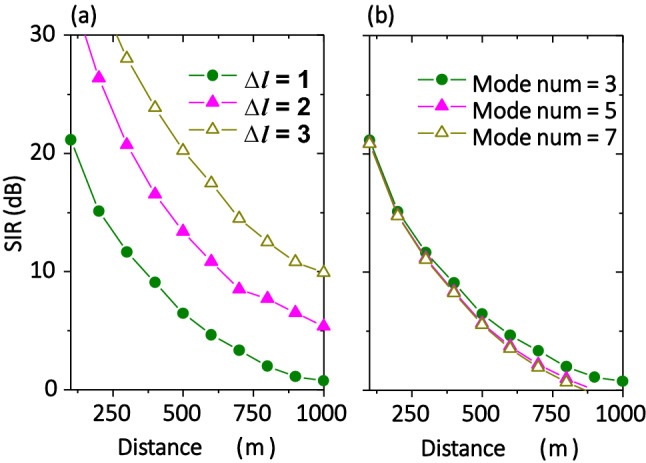
Signal-to-Interference ratio performance for multiple multiplexed OAM beams. Systems with (**a**) 3-muxed OAM modes with mode spacing from 1 to 3 (OAM beams of [+ 3, + 4, + 5]; [+ 2, + 4, + 6]; and [+ 1, + 4, + 7] are transmitted, respectively), and (**b**) 3-to-7-muxed OAM modes with a mode spacing of 1 (OAM beams of [+ 3, + 4, + 5]; [+ 2, + 3, + 4, + 5, + 6]; and [+ 1, + 2, + 3, + 4, + 5, + 6, + 7] are transmitted, respectively). The parameters are set as ${C}_{n}^{2}$ = 1 × 10^–11^ m^-2/3^, *w*_*0*_ = 0.1 m and *f* = 0.5 THz. Scales in *y*-axis in (**a**,**b**) are the same.

We study the XT performance of various THz OAM links without simultaneously transmitting multiple beams. Multiplexing OAM beams might further degrade the system performance under turbulence. Here, we analyze the SIR performance of THz links that multiplex various sets of multiple OAM beams (Fig. 7). SIR is defined as the ratio of the signal power to the crosstalk from the other channels, where the signal power is the received power on transmitted mode, and the noise is the total power leakage from the other channels to the transmitted mode. We simulate the SIR performance when varying: (i) mode spacing from 1 to 3 with 3 OAM modes multiplexed; and (ii) the number for multiplexed modes from 3 to 7 with a mode spacing of 1. For the THz links with 3 OAM modes multiplexed, the SIR is enhanced by ~ 5–7 dB with mode spacing increased by 1. This might be mainly due to the fact that there is a smaller power leaked to higher-neighouring modes under turbulence. Moreover, for THz links with a mode spacing of 1, the SIR decreases by ~ 0.5–1 dB with the number of modes increased by 1. This small value change might be explained by that the channel crosstalk from OAM modes with a mode spacing of > 1 is small (such as < − 15 dB) when the phasefront distortion under turbulence is not strong.

### Influence of link and system parameters on XT performance

#### Turbulence condition

**Figure 8:**
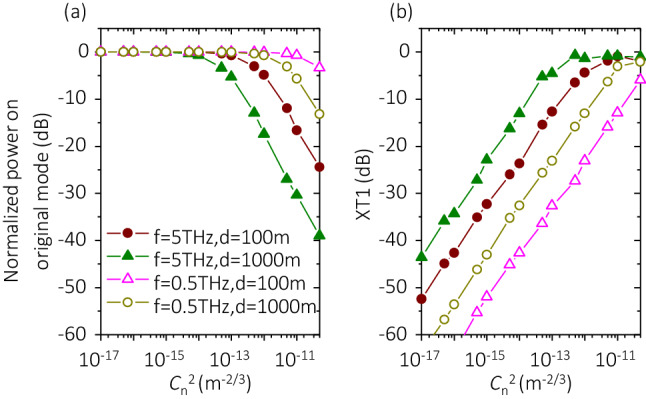
Effects of varying ${C}_{n}^{2}$ on system performance. 100-m links with (**a**) ${C}_{n}^{2}$ = 0 m^−2/3^, (**b**) ${C}_{n}^{2}$ = 1 × 10^–13^ m^−2/3^, and (**c**) ${C}_{n}^{2}$ = 1 × 10^–11^ m^−2/3^. Other parameters are set as *w*_*0*_ = 1 m, *f* = 5 THz, and OAM + 4 is transmitted. Legends and scales in *y*-axis in (**a**–**c**) are the same. *XT* crosstalk.

We show the dependence of the XT performance in THz OAM links with fixed turbulence conditions. To further study the effect of turbulence conditions, we investigate the XT performance in OAM THz links with different ${C}_{n}^{2}$. Figure 8 shows the signal power and XT1 of THz-OAM links with different distances and carrier frequencies. As shown in Fig. 8a, when we transmit OAM with *w*_*0*_ = 1 m at *f* = 5 THz in a 1-km link, the signal power decreases by ~ 40 dB and XT1 increases by ~ 42 dB, with ${C}_{n}^{2}$ varied from 5 × 10^–18^ to 5 × 10^–11^ m^−2/3^. This could be because the value of *r*_*0*_ decreases when ${C}_{n}^{2}$ is increased (see Eq. (2)), which results in a larger value of *D*/*r*_*0*_ and thus a stronger distortion. Moreover, the results in Fig. 8b show that for the cases when an OAM + 4 with *w*_*0*_ = 1 m propagates through a 1-km link at *f* = 5 THz, or at *f* = 0.5 THz, XT1 can remain < − 15 dB when ${C}_{n}^{2}$ < 1 × 10^–14^ m^−2/3^, and ${C}_{n}^{2}$ < 5 × 10^–13^ m^−2/3^, respectively.

#### Limited aperture size

**Figure 9:**
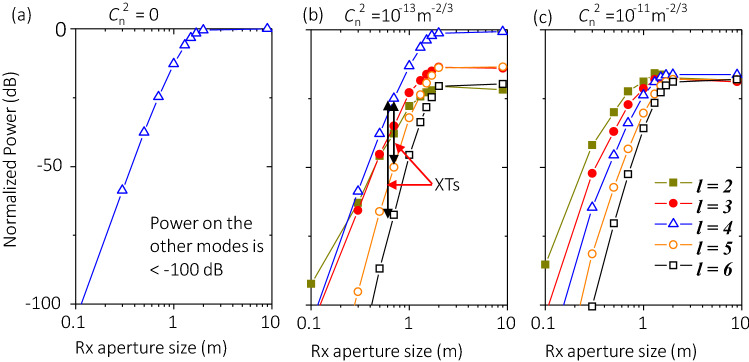
Effects of varying the aperture size on normalized power distribution on different OAM modes. 100-m links with (**a**) ${C}_{n}^{2}$ = 0 m^−2/3^, (**b**) ${C}_{n}^{2}$ = 1 × 10^–13^ m^−2/3^, and (**c**) ${C}_{n}^{2}$ = 1 × 10^–11^ m^−2/3^. Other parameters are set as *w*_*0*_ = 1 m, *f* = 5 THz, and OAM + 4 is transmitted. Legends and scales in *y*-axis in (**a**–**c**) are the same. *XT* crosstalk.

The Rx might not recover all the power of the received OAM beam due to the limited aperture size. In this simulation, we explore the effect of limited Rx aperture size on the XT performance. We consider the cases for 100-m links with ${C}_{n}^{2}$ = 0 (no turbulence), 1 × 10^–13^, and 1 × 10^–11^ m^−2/3^. Other parameters are set as *w*_*0*_ = 1 m, and *f* = 5 THz, and OAM + 4 is transmitted. We find through simulations that: (i) without turbulence, limited aperture size tends to only result in power loss on the transmitted mode, and the power leakage to neighbouring OAM modes remains < − 100 dB (Fig. 9a); (ii) with both calm weather (${C}_{n}^{2}$= 1 × 10^–13^ m^−2/3^) and bad weather (${C}_{n}^{2}$= 1 × 10^–11^ m^-2/3^), limited aperture size (< 2 m) leads to decreased signal power, decreased XT to higher-order modes, and increased XT to lower-order modes (the power difference among the blue curve and other curves in Fig. 9b,c). As an example, the XT from transmitted OAM + 4 to OAM + 3 increases by ~ 4 dB while the XT to OAM + 5 decreases by ~ 6 dB, when the Rx size decreases from 2 to 1 m, as shown in Fig. 9c. The reason might be that lower-order OAM modes diverge slower such that these modes have smaller beam sizes at the receiver side. As a result, a limited Rx could receive more power on lower OAM modes.

#### Misalignment

**Figure 10:**
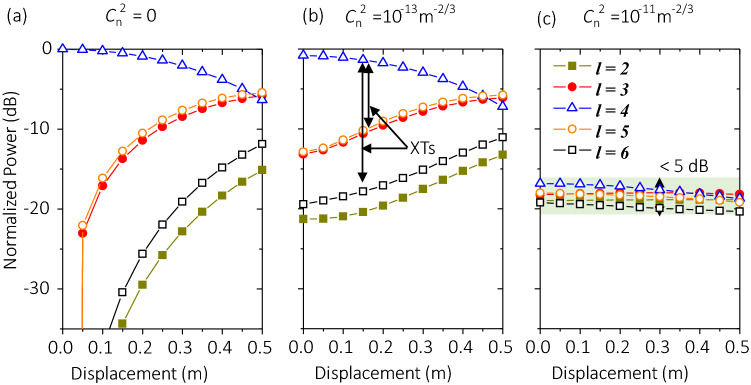
Effects of varying the displacement on the normalized power distribution on different OAM modes. 100-m links with (**a**) ${C}_{n}^{2}$ = 0 m^−2/3^, (**b**) ${C}_{n}^{2}$ = 1 × 10^–13^ m^−2/3^, and (**c**) ${C}_{n}^{2}$ = 1 × 10^–11^ m^-2/3^. Other parameters are set as *w*_*0*_ = 1 m, *f* = 5 THz, and OAM + 4 is transmitted. Legends and scales in *y*-axis in (**a**–**c**) are the same. *XT* crosstalk.

Moreover, the displacement might also occur at the receiver side. This could be considered as an additional lateral shift to the phasefront of the received beam, which might result in channel crosstalk to the system. In this simulation, we explore the effect of varying the displacement between Tx and Rx on the XT performance, as shown in Fig. 10. We consider the cases for 100-m links with ${C}_{n}^{2}$ = 0 (no turbulence), 1 × 10^–13^, and 1 × 10^–11^ m^−2/3^. Other parameters are set as *w*_*0*_ = 1 m, and *f* = 5 THz, and OAM + 4 is transmitted. We assume that the Rx aperture size is larger than the received beam. Simulation results show that: (i) under all three turbulence conditions, displacement leads to decreased signal power and increased XT to neighbouring modes; however (ii) with bad weather (${C}_{n}^{2}$ = 1 × 10^–11^ m^−2/3^), the signal power decreases slower and the XT increases slower as displacement is varied from 0 to 0.5 m. For example, both the signal power and XT fluctuate in a small range of < 5 dB. This might be due the following: (i) the power of the received beam is almost averagely leaked to other modes under bad weather; and (ii) even if there is a displacement between the Tx and Rx, it does not change the strength of the phasefront distortion *D*/*r*_*0*_ too much, leading to a low power leakage to other modes.

## Discussion

We study through simulations modal coupling and crosstalk due to turbulence and divergence effects on free-space THz communication links when using multiple OAM beams. The influences of various beam parameters and link system parameters on the signal power loss and XT are considered. We find through simulation that the divergence and turbulence effects in the THz regime are different from those in the mm-wave and optical regime. The divergence effect tends to be more important in free-space mm-wave OAM links; however, optical OAM links tend to be mainly limited by the turbulence effect. Our simulations show that both the divergence and turbulence effects tend to be important in THz OAM links, which might be carefully considered to achieve a low modal coupling and crosstalk performance.

The following points are also worth mentioning: (i) We only consider the power leakage to other OAM modes, namely, Laguerre-Gaussian (LG_*l,p*_) modes with a zero *p* value. There might also be power leaked to neighbouring LG_*l,p*_ modes with non-zero *p* values; (ii) We focus on atmospheric turbulence and explore atmospheric attenuation a little bit. Atmospheric dispersion also affects THz communications, leading to different phase delays at different frequencies^21^; (iii) We assume atmospheric turbulence is time invariant. However, in a practical THz OAM link, many factors including air pressure variation and wind velocity might result in fluctuation of both amplitude and phase of THz beams^25^; (iv) We focus on link distances of 100 m to 1 km. For a longer link distance of > 1 km, a lower frequency might be needed due to high atmospheric attenuation at THz of > 0.5 THz. However, there might be more power loss due to that lower-frequency beam diverges faster. For a shorter link distance of < 100 m, smaller Tx and Rx aperture sizes of < 10 cm might be used. Since smaller beam diverges faster, it might affect the signal power and XT in a different manner; (v) We only consider the cases of transmitting OAM beams with positive OAM values. The cases of transmitting OAM beams with negative OAM values could have similar properties regarding both the diffraction and turbulence effects. The crosstalk from the positive OAM value to the negative one could be smaller than that from the positive OAM value to the positive one. This might be due to that the OAM value difference is higher in the positive-to-negative case, and thus there is less power leakage from the positive one to the negative one^12,13^.

## Methods

### Simulation model considerations

The refractive index structure constant ${C}_{n}^{2}$ could be used to quantify the turbulence strength, which represents the atmospheric refractive index inhomogeneities^28^. This is because the value of ${C}_{n}^{2}$ could be in the range of 1 × 10^–15^ to 1 × 10^–11^ m^−2/3^, which can represent lower (i.e., relatively weaker) to higher (i.e., relatively stronger) turbulence strengths^17,20^. Moreover, the Rytov variance ${\sigma }_{R}^{2}$ can physically represent the turbulence fluctuation, *i.e.*, the fluctuation in the intensity of the propagating beam^28^. This is because the value of ${\sigma }_{R}^{2}$ is dependent on the atmospheric refractive index inhomogeneities (i.e., ${C}_{n}^{2}$), beam wavelength, and the link distance^28^. The weaker, moderate, and stronger atmospheric turbulence fluctuations can be therefore associated with ${\sigma }_{R}^{2}<1$, ${\sigma }_{R}^{2}\sim 1$, and ${\sigma }_{R}^{2}>1$, respectively^28^.

There have been different simulation models used to describe the electromagnetic wave transmission through turbulent atmosphere^28–31^. Depending on the value of ${\sigma }_{R}^{2}$, these models could be classified as weaker, moderate, and stronger turbulence fluctuation theories^28^. For example, the Kolmogorov spectrum model^28^, the Gamma-Gamma distribution model^29^, and the K distribution model^31^ have been used to simulate under the weaker (${\sigma }_{R}^{2}<1$), moderate (${\sigma }_{R}^{2}\sim 1$), and stronger (${\sigma }_{R}^{2}>1$) turbulence fluctuation conditions, respectively. Specifically, by using these models under different turbulence fluctuation conditions, the Kolmogorov spectrum model has been used to predict the received beams’ spectrum with an error of less than 1 × 10^–2^ ^28^, and the Gamma-Gamma distribution model and K distribution model have been used to predict the received beam’s intensity distribution with an error of less than 1 × 10^–3^ ^30^.

In this study, we use the Kolmogorov spectrum model to simulate the turbulence effects on THz OAM beams. This model does not fit well for the stronger turbulence fluctuation (${\sigma }_{R}^{2}>1$), because the effect of multiple self-interference could lead the beam to be less coherent^28^. The range of ${\mathrm{C}}_{\mathrm{n}}^{2}$ in this study is selected to be 1 × 10^–17^ to 1 × 10^–11^ m^−2/3^, which represents the weaker to the stronger turbulence strength. However, we show by calculation that all the cases in this work are under the weaker turbulence fluctuation condition, i.e., ${\sigma }_{R}^{2}<1$.

According to the Kolmogorov spectrum model, the relationship between ${C}_{n}^{2}$ and ${\sigma }_{R}^{2}$ can be written as^28^

1 $${\sigma }_{R}^{2}=1.23{C}_{n}^{2}{k}^{7/6}{L}^{11/6}$$

where $k=2\uppi /\lambda$ is the wave number and $L$ is the propagation length. Based on Eq. (1), the maximum values of ${\sigma }_{R}^{2}$ for Figs. 3, 4, 5, 6, 7, 8, 9, 10 (except for the concept Figs. 1 and 2) are calculated to be around 0.43, 0.33, 0.05, 0.19, 0.19, 0.19, 0.04, and 0.04, respectively. All of these ${\sigma }_{R}^{2}$ values satisfy the weaker turbulence fluctuation condition, i.e., ${\sigma }_{R}^{2}<1$. Therefore, we utilize the Kolmogorov spectrum model by transmitting THz beams through multiple phase plates, which has been reportedly used for simulating the weaker turbulence fluctuation^28^.

We note that due to that the THz beam has a relatively longer wavelength when compared with the optical beam, the value of ${\sigma }_{R}^{2}$ could be < 1 even with a larger value of ${C}_{n}^{2}$ of 1 × 10^–11^ m^−2/3^. Our model might not be appropriate if we further increase (i) the frequency beyond 10 THz, or (ii) the distance beyond 1 km, where a stronger turbulence fluctuation condition may be satisfied. Under these conditions, the value of ${\sigma }_{R}^{2}$ could be > 1. Under the stronger turbulence fluctuation condition, other models such as the K distribution model might be suitable^31^.

### Simulation details

We first build a model to emulate the turbulence, which is based on Kolmogorov turbulence theory^13^. Atmospheric turbulence leads to a refractive index fluctuation, and thus distorts phasefront of beams propagating in atmosphere. The refractive index fluctuation can be described by a structure function *D*_*n*_ as^12,13^

2 $${D}_{n}(\Delta r)=6.88{(\frac{\Delta r}{{r}_{0}})}^{5/3}$$

where *∆r* is the distance between two points in atmosphere, *r*_*0*_ is the Fried parameter which is commonly designated as the coherence length of atmospheric turbulence. The *r*_*0*_ value can be driven from atmospheric structure constant ${C}_{n}^{2}$ by using^12,13^

3 $${r}_{0}={(0.423{k}^{2}{C}_{n}^{2}L)}^{-3/5}$$

where *k* = 2π/ *λ* is the wave number and *L* is the propagation length.

The value of ${C}_{n}^{2}$ is related to the atmospheric temperature and humidity distributions^17^. In general, the variation of the relative temperature and humidity are about ± 0.6% and ± 0.1 °C for calm weather condition, and about ± 2.7% and ± 1.0 °C for unstable weather conditions^20^. Given the variation values, the values of ${C}_{n}^{2}$ are estimated at around 1 × 10^–13^ and 1 × 10^–11^ m^−2/3^, which can represent relatively moderate and stronger turbulence strength, respectively^17,20^. Therefore, we refer ${C}_{n}^{2}$ of 1 × 10^–13^ and 1 × 10^–11^ m^−2/3^ as for “calm” and “bad” weather conditions for THz beams in our paper, respectively. We assume ${C}_{n}^{2}$ remain the same during propagation in atmosphere.

Each emulated phase plate can be described by the turbulence-induced phasefront distortion, which is driven from the refractive index fluctuation in Eq. (2). Based on the Kolmogorov theory^13^, the spectrum of fluctuations in the refractive index can be expressed as^28^

4 $${\phi }_{n}(\kappa )=0.023{r}_{0}^{-5/3}{k}^{-11/3}$$

where $\kappa =$ $2\pi ({f}_{x}\bullet \widehat{x}+{f}_{y}\bullet \widehat{y})$ is the angular spatial frequency in rad/m, and ${r}_{0}$ is the Fried parameter. Thus, the phase distribution of turbulent atmosphere can be obtained according to Eq. 5 as^28^

5 $$\psi (\kappa )=\mathrm{exp}(i{\phi }_{n}(\kappa ))$$

where $\psi (\kappa )$ is the phase distribution of each phase plate in our simulation model. Moreover, the spacing between neighboring emulated phase plates is generally between 1 to 100 m^28^. In our simulation model, time cost is proportional to the number of phase plates, and thus we take it as 10 (phase plate spacing is 100 m) to save the time cost. We assume that the atmospheric structure constant ${C}_{n}^{2}$ remains the same at different distances. The input OAM beams are a subset of LG_*l,p*_ beams with zero *p* values. The size of the simulation screen is 20 m, and *N* is 2000.

### Assumptions

For the convenience of analysis, the following assumptions are made:

(i). We focus on atmospheric turbulence and discuss atmospheric attenuation a little bit only in Fig. 6c,d. However, we note that atmospheric attenuation is mainly due to the absorption of water vapor and oxygen in THz domain^16^, and it could be even > 100 dB/km as *f* is > 0.5 THz^16,21^. Although atmospheric attenuation is one important effect in THz communications, we still assume there is no atmospheric attenuation and neglect its effects on the system performance, except for Fig. 6c,d. The reasons are as follows:
  - The absorption could be extremely high (> 100 dB) for a link with a relatively longer distance (up to 1 km in this study) at the higher frequency (> 0.5 THz). We might not be able to clearly determine the influence of beam parameters and system parameters on the XT performance if we consider the absorption. As the example in Fig. 6c shows, different curves are close to each other and thus it might be hard to distinguish the power distribution on different OAM orders; and
  - Moreover, when considering the absorption, the received power on different OAM modes would decrease by the same amount while XTs to neighbouring modes would remain almost unchanged;
(ii). Similarly, in order to isolate the effect of limited Tx/Rx aperture size, we assume the aperture size is larger than transmitted/received OAM beams, except for Fig. 9. However, when the Tx/Rx aperture size is smaller than transmitted/received beams, the power on the transmitted/received mode would decrease;
(iii). The input power of each transmitted OAM beam is 0 dBm;
(iv). The transmitter aperture is always larger than the input beam size, and we assume that it does not affect the intensity and phase profiles of the input beam. If the transmitter aperture is less than the input beam size, the mode purity of the transmitted OAM beams would decrease;
(v). We only consider the case of a single-polarized system. We note that there is no obvious difference in the turbulence effect for orthogonal polarizations in optical domain^1,12^. The turbulence effect on a double-polarized THz system might need further exploration.
(vi). Each data point in all figures with non-zero ${C}_{n}^{2}$ is calculated by averaging 50 simulation iterations.

### Details of parameter definitions

For the convenience of analysis, the following definitions about OAM beams are used:

(i) Electric fields of both the transmitted and received beams are described by a subset of LG_*l,p*_ beams with a zero *p* value, which is defined as^8^
  7 $${LG}_{l,p}\left(r,\theta ,z,\omega \right)=\frac{{C}_{l,p}^{LG}}{w\left(z\right)}{\left(\frac{r\sqrt{2}}{w\left(z\right)}\right)}^{\left|l\right|}{\mathrm{exp}}\left(-\frac{{r}^{2}}{{w}^{2}\left(z\right)}\right){L}_{p}^{\left|l\right|}\left(\frac{{2r}^{2}}{{w}^{2}\left(z\right)}\right)\times {\mathrm{exp}}\left(-i\left(k\frac{{r}^{2}}{2R\left(z,\omega \right)}+l\uptheta +kz-\psi \left(z,\omega \right)\right)\right)$$
  where ${L}_{p}^{\left|l\right|}$ are the generalized Laguerre polynomials, ${C}_{l,p}^{LG}$ are the required normalization constants, $w\left(z\right)$ is the beam size at a distance of *z*, $R\left(z,\omega \right)=z(1+{({z}_{R}(\omega )/z)}^{2})$ is the wavefront radius, where ${z}_{R}(\omega )$ is the Rayleigh range, $k$ is the wave number, $\omega$ is the angular frequency, $\left(r,\theta ,z\right)$ represents the cylindrical coordinate, and $\psi \left(z\right)$ is the Gouy phase and equals $\left(\left|l\right|+2p+1\right)\mathrm{arctan}\left(z/{z}_{R}(\omega )\right)$;
(ii) The beam size (or diameter) of an OAM beam with an OAM order of *l* after propagation to a distance of *z* can be written as^10,26^
  8 $$D={w}_{0}\sqrt{\left|l\right|+1}\times \sqrt{1+{\left(\frac{2z}{k{{w}_{0}}^{2}}\right)}^{2}}$$
  where ${w}_{0}$ is the waist at the distance of 0. Except for Fig. 5 where the value of ${w}_{0}$ is varied, OAM beams in all the other cases have different beam diameter $D$ but the same beam waist ${w}_{0}$, which is equal to the beam waist of a fundamental Gaussian beam;
(iii) We calculate the OAM spectrum of the received beam as the normalized power weight coefficient of each OAM mode as^27^
  9 $${{|C}_{l}|}^{2}={\left|\iint {E}_{1}\left(x,y\right){E}_{2}^{*}\left(x,y\right)dxdy \right|}^{2}$$
  where ${E}_{1}\left(x,y\right)$ is the electric field of the received beam, and ${E}_{2}\left(x,y\right)$ is the electric field of a pure ${\mathrm{LG}}_{l,0}$ beam with same beam parameters at the receiver aperture. Both ${E}_{1}\left(x,y\right)$ and ${E}_{2}\left(x,y\right)$ are normalized, namely, ${\left|\iint {E}_{i}\left(x,y\right){E}_{i}^{*}\left(x,y\right)dxdy \right|}^{2}=1$, where *i* = 1 or 2.

## Data Availability

All data, theory details, simulation details that support the findings of this study are available from the corresponding authors on reasonable request.
